# Beyond Dimorphism: Body Size Variation Among Adult Orangutans Is Not Dichotomous by Sex

**DOI:** 10.1093/icb/icad015

**Published:** 2023-04-14

**Authors:** Alexandra E Kralick, Caitlin A O'Connell, Meredith L Bastian, Morgan K Hoke, Babette S Zemel, Theodore G Schurr, Matthew W Tocheri

**Affiliations:** Department of Anthropology, University of Pennsylvania, Philadelphia, PA 19104, USA; Department of Anthropology, University of Pennsylvania, Philadelphia, PA 19104, USA; Department of Anthropology, Rutgers, the State University of New Jersey, New Brunswick, NJ 08901, USA; Proceedings of the National Academy of Sciences, Washington, DC 20001, USA; Department of Anthropology & Carolina Population Center, University of North Carolina at Chapel Hill, Chapel Hill, NC 27599, USA; Division of Gastroenterology, Hepatology and Nutrition, The Children's Hospital of Philadelphia, Philadelphia, PA 19104, USA; , Department of Pediatrics, The University of Pennsylvania Perelman School of Medicine, Philadelphia, PA 19104, USA; Department of Anthropology, University of Pennsylvania, Philadelphia, PA 19104, USA; Department of Anthropology, Lakehead University, Thunder Bay, ON P7B 5E1, Canada; Human Origins Program, Department of Anthropology, National Museum of Natural History, Smithsonian Institution, Washington, DC 20013, USA; Australian Research Council Centre of Excellence for Australian Biodiversity and Heritage, University of Wollongong, Wollongong NSW 2522, Australia

## Abstract

Among extant great apes, orangutans are considered the most sexually dimorphic in body size. However, the expression of sexual dimorphism in orangutans is more complex than simply males being larger than females. At sexual maturity, some male orangutans develop cheek pads (flanges), while other males remain unflanged even after becoming reproductively capable. Sometimes flange development is delayed in otherwise sexually mature males for a few years. In other cases, flange development is delayed for many years or decades, with some males even spending their entire lifespan as unflanged adults. Thus, unflanged males of various chronological ages can be mistakenly identified as “subadults.” Unflanged adult males are typically described as “female-sized,” but this may simply reflect the fact that unflanged male body size has only ever been measured in peri-pubescent individuals. In this study, we measured the skeletons of 111 wild adult orangutans (*Pongo* spp.), including 20 unflanged males, 45 flanged males, and 46 females, resulting in the largest skeletal sample of unflanged males yet studied. We assessed long bone lengths (as a proxy for stature) for all 111 individuals and recorded weights-at-death, femoral head diameters, bi-iliac breadths, and long bone cross-sectional areas (CSA) (as proxies for mass) for 27 of these individuals, including seven flanged males, three adult confirmed-unflanged males, and three young adult likely-unflanged males. ANOVA and Kruskal–Wallis tests with Tukey and Dunn post-hoc pairwise comparisons, respectively, showed that body sizes for young adult unflanged males are similar to those of the adult females in the sample (all *P* ≥ 0.09 except bi-iliac breadth), whereas body sizes for adult unflanged males ranged between those of adult flanged males and adult females for several measurements (all *P* < 0.001). Thus, sexually mature male orangutans exhibit body sizes that range from the female end of the spectrum to the flanged male end of the spectrum. These results exemplify that the term “sexual dimorphism” fails to capture the full range of variation in adult orangutan body size. By including adult unflanged males in analyses of body size and other aspects of morphology, not as aberrations but as an expected part of orangutan variation, we may begin to shift the way that we think about features typically considered dichotomous according to biological sex.

## Introduction

Orangutans are said to be the most sexually dimorphic in body size of all extant great ape taxa (Smith and Pilbeam 1980; Galdikas 1985a; Morbeck and Zihlman 1988; Uchida 1992; Knott and Kahlenberg 2007). Adult males with fully developed secondary sex characteristics typically weigh more than twice as much as adult females (Schultz 1969; Eckhardt 1975; Leutenegger and Kelly 1977; Cant 1987; Markham and Groves 1990; Leigh and Shea 1995; Delgado and van Schaik 2000; Schuppli et al. 2021; but see Smith and Jungers 1997). However, some sexually active male orangutans are described as “adult-female sized” (Utami Atmoko et al. 2009a), which can be distinguished from the other males by the absence of secondary sex characteristics, such as large visible bidiscoid pads (i.e., flanges) on the face and a laryngeal throat pouch for mate calling (Utami-Atmoko and van Hooff 2004; Kuze et al. 2005; Rayadin and Saitoh 2009; Pradhan et al. 2012; Marty et al. 2015). Our understanding of the behavior and biology of male orangutans without flanges has changed dramatically over the last 50 years, but body size variation in these unflanged males has yet to be re-evaluated. Thus, the aim of this study was to document and describe variation in several skeletal correlates of body size in a sample of mature orangutan skeletons and skins.

The wide range of variation in adult male orangutan body shape and size has resulted in considerable confusion among researchers. In the 1800s, long before the terms flanged and unflanged were used, Alfred Russel Wallace identified these two male morphs as separate species (Wallace 1856a, 1856b), an idea that may go back to Petrus Camper in the 1700s (Meijer 1991, 2014). One hundred and twenty years later, instead of different species, these males were thought to represent different stages of development the same individual went through, with males who displayed secondary sex characteristics defined as adults and those who did not defined as subadults (MacKinnon 1974; Rijksen 1978; Mitani 1985; Galdikas 1985a, 1985b; Schürmann and van Hooff 1986; Utami et al. 1997; Fox 2002). More specifically, it was thought that, around age 8 or 10 years old, an orangutan male would shift from a juvenile stage of development to a “subadult” one during which they were sexually active but lacked secondary sex characteristics until reaching full adulthood around 14 or 15 years of age (MacKinnon 1974; Rijksen 1978), which is the average age of puberty for male orangutans (MacKinnon 1974; Morbeck and Zihlman 1988; Wich et al. 2004; Knott and Kahlenberg 2007). Although puberty itself is inconsistently defined, sometimes meaning the onset of secondary sexual characteristic development and at other times the start of adolescence (see Russon 2006 versus Knott and Kahlenberg 2007), adolescence is defined as weaned individuals showing some sexual activity between ages 8–14 and 10–14 years (Wich et al. 2004; Takeshita 2019). Thus, the term “subadult” indicated that males were in transition to full adulthood (Utami-Atmoko and van Hooff 2004).

Subsequently, male orangutans without flanges that were sexually active and older than 14–15 years of age were found, including individuals up to 17 or 18 years old in captivity and even 19 or 20 years old in the wild (Galdikas 1985a). Not long afterward, other unflanged males were observed to be 10 years or more past becoming sexually active, suggesting that some male orangutans remained in this “subadult” phase for 50–60% of their estimated reproductive lifespan (te Boekhorst et al. 1990). Despite being fully adult, however, these males were still referred to as “subadults.” It was not until the 21st century that males who remained without flanges into their 30s, or possibly for their entire lifetimes, were discovered (Utami et al. 2002; Utami Atmoko and van Hooff 2004; Wich et al. 2004; Knott and Kahlenberg 2007). This discovery led to the insight that the term “subadult” was misleading and that these males should be considered adults (Utami Atmoko and van Hooff 2004). Accordingly, studies began to refer to these sexually active males without flanges not as “subadults” but instead as unflanged males (e.g., Kuze et al. 2005; Rayadin and Saitoh 2009; Pradhan et al. 2012; Marty et al. 2015), avoiding the ambiguity inherent in the term “subadult” (Setchell and Lee 2004; Utami Atmoko and van Hooff 2004). Following this practice, we also avoid using “subadult” in this context and instead adopt the convention used by Kralick and McGrath (2021) and refer to sexually active males with unflanged faces in their adolescent years as “young adult unflanged males.” In this study, we refer to adult unflanged males and young adult unflanged males collectively as “unflanged males”. This feature is not necessarily dichotomous, though, as flange development occurs over a widely variable period of time (Maggioncalda 1999) and some older individuals have diminished flanges, known as “past prime” males (Knott 2009).

**Table 1.** Size of the entire sample across 13 museums in 5 countries broken up by sex and flanging status and by types of data available

				Sample Size						Types of data available				
				Adult and young adult males				Adult females		Long bone length	CSA	Femoral head diameter	Bi-Iliac breadth	Weight
				Unflanged		Flanged								
Museum	Acronym	Location	Country	Confirmed	Likely	Confirmed	Likely							
Penn Museum, University of Pennsylvania	Penn Museum	Philadelphia	USA	0	1	0	0	2		Yes	Yes	Yes	No	No
Academy of Natural Sciences of Drexel University	ANSP	Philadelphia	USA	0	1	1	2	2		Yes	Yes	Yes	No	No
Museum of Comparative Zoology—Harvard University	MCZ	Cambridge	USA	0	0	1	1	3		Yes	Yes	Yes	No	No
Zoologisches Museum/Museum fur Naturkunde	MfN	Berlin	Germany	0	0	0	0	1		Yes	No	No	No	No
American Museum of Natural History	AMNH	New York	USA	0	3	1	1	2		Yes	Yes	Yes	No	No
Smithsonian Institution National Museum of Natural History	USNM	Washington DC	USA	2	3	6	2	14		Yes	Yes	Yes	Yes	Yes
Natural History Museum	NHM	London	UK	0	2	2	10	2		Yes	Yes	Yes	No	No
Field Museum of Natural History	FMNH	Chicago	USA	0	2	3	2	2		Yes	Yes	No	No	No
Denver Museum of Nature and Science	DMNS	Colorado	USA	0	0	1	1	4		Yes	No	No	No	No
State Museum of Natural History	SMNS	Stuttgart	Germany	0	2	1	0	1		Yes	No	No	No	No
Zoologische Staatssammlung München	ZSM	Munich	Germany	0	2	1	3	5		Yes	No	No	No	No
Anthropologisches Institüt und Museum der Universität Zürich	UZH	Zurich	Switzerland	1	1	0	4	5		Yes	No	Yes	No	No*
Muséum National d'Histoire Naturelle	MNHN	Paris	France	0	0	0	2	3		Yes	No	No	No	No
			Total	3	17	17	28	46						
			Total	20		45								

* one specimen at UZH was discovered to be the same individual at USNM, traded by Adolf Schultz, so weight was available

Previous studies that used the convention of describing unflanged males as being “female-sized” only reported sizes for peri-pubescent males around 10–15 years old (i.e., young adult unflanged males) and not adult unflanged males (Galdikas 1985a; Delgado and van Schaik 2000; Fox 2002; Rayadin and Saitoh 2009; Manduell et al. 2011; Banes et al. 2015). Body sizes of adult unflanged males remain relatively unexplored, and it is possible that only young adult unflanged males are “female-sized.” Growth may continue during these ages, such that adult unflanged males are larger than females. For example, males up to 19 or 20 years old that still lack secondary sex characteristics appear to be larger in body size when compared with younger ones (Galdikas 1985). Nine Bornean young adult unflanged males (*Pongo pygmaeus*) measured at an orangutan rehabilitation center also suggested that those who remain in the unflanged state for more time are heavier on average than adult females but lighter than flanged males (Prasetyo 2019). Among these unflanged males, those 12–14 years old weighed between 40.0 and 53.6 kg, whereas those 14–16 years old weighed between 49.6 and 82.5 kg, values that are more similar to the 55.0–88.9 kg range for flanged males age 12–20 years old (Prasetyo 2019). Similarly, an arm length of 395.9 mm for one wild unflanged male that was 15 years old was well within the arm length range of flanged males (346.7–550.1 mm) (Brown et al. 2022). Finally, the estimated muscle mass of unflanged males who were considered fully adult based on overall size and appearance was not significantly different from flanged males (O'Connell et al. 2021). Moving forward, we do not follow the convention of saying “female-sized” but instead refer to how unflanged males relate to the adult females in the sample, whether they fail to overlap in size or are statistically indistinguishable in size.

These findings suggest that more studies include measurements of adult males with delayed expression of secondary sex characteristics beyond the range for puberty to gain insight into the regulation of growth, pubertal development, and sexual dimorphism. Unfortunately, data for unflanged male body size are scarce, especially for fully adult males, since males all develop flanges by age 17 or 18 years in captivity (Emery Thompson et al. 2012; Pradhan et al. 2012), and measuring body size in wild populations is inherently challenging, as darting and capturing wild apes for research is generally considered to be unethical and not permissible (Brown et al. 2022). Thus, natural history collections of wild orangutans offer an important opportunity to measure features that correlate with body size variation and, in turn, re-evaluate patterns of sexual dimorphism in this Asian great ape. These collections also provide the advantage of estimating an individual’s age through dental development and wear patterns, whereas estimating age in the wild can be more challenging (van Noordwijk et al. 2018). Here, we define body size as stature and mass (Kurki et al. 2010). We also measured postcranial features, which have a more direct relationship with body size than cranial dimensions (Auerbach and Ruff 2004). Body mass is often estimated from two-dimensional bone measurements such as femoral head diameter and bi-iliac breadth (e.g., Ruff et al. 2005; Kurki et al. 2010; Ruff 2010). Similarly, stature is often estimated from maximum femoral length (Jungers 1988; Hens et al. 1998). We further used long bone cross-sectional dimensions, which are strong predictors of body mass in primates (Burgess et al. 2018). Using these measurements, we address two primary questions. First, how do the body masses and various skeletal size measures of unflanged males compare to those of flanged males and adult females? Second, are body mass and skeletal size in adult orangutans binary (i.e., large males and small females) or are they situated on a more complex spectrum?

## Methods

We studied the skeletons of 111 adult orangutans (*Pongo* spp.) collected in the wild from the 1800s to the mid-1900s and now curated in 12 museums in five countries (Table 1). Age-at-death was estimated by patterns of dental development and occlusal wear ( Kralick 2018; Kralick and McGrath 2021). Individuals were considered either adults if one or more of their permanent third molars was fully erupted in occlusion with roots fully developed (Schultz 1941) or young adults if the development of the roots of one or more of their permanent third molars was in progress. Sex was determined from museum records and confirmed using measurements of permanent canine height (see Kralick and McGrath 2021). Male skeletons were classified as flanged or unflanged by measuring the face of an associated skin from the eye to the ear (9.3–18.0 cm vs. 8.0–9.0 cm, respectively).

Males in the process of flanging or “past prime” males whose flanges have diminished in size (Knott 2009) are not considered here because of the obvious challenges of identifying such individuals from skeletal evidence alone. We confirmed in prior studies (Kralick and McGrath 2021; Kralick et al. in revision) that flanged and unflanged males show distinct patterns of limb bone and dental development (see Table 2). Thus, when no associated skin was available, male skeletons were identified as likely-flanged or likely-unflanged based on whether the limb bone epiphyses were fused or unfused, respectively, in combination with fully erupted permanent dentition and “male-sized” canines (>19 mm in height) (Kralick and McGrath 2021).

**Table 2.** Criteria for establishing categories of age and flanging status for all individuals

	Flanging status		Sex	Age	
Category	Skin of face (measurement eye to ear in cm)	Long bone epiphyses	Canine height (measurement in mm)	Dentition	Dental wear
Adult confirmed-flanged male	Flanged (size range 9.25–18)	Closed	Tall (range 19–28)	Adult: all teeth including M3 in occlusion (root of M3 fully developed in CT scan if available)	Range of adults from almost none to extensive
Adult likely-flanged male	No skin				
Adult confirmed-unflanged male	Unflanged face (size range 8–9)	Open or fusing			
Adult likely-unflanged male	No skin				
Adult flanging male	Skin developing flanges	Closed			
Adult female	Either no skin or small face (size range 6.75–9)		Small (range 12–17)		
Young adult confirmed-unflanged male	Unflanged face (size range 8–9)	Open or fusing	Tall (range 19–28)	Young adult: all teeth including M3 in occlusion but M3 root is developing	Little to none
Young adult likely-unflanged male	No skin				

Maximum long bone lengths for femora, tibiae, radii, ulnae, and humeri were measured using an osteometric board. If epiphyses were unfused, then they were articulated prior to measurement. Maximum long bone lengths were measured in the entire sample, while femoral head diameters, bi-iliac breadths, and long bone cross-sectional areas (CSAs) were measured in a subsample of 27 individuals from the Smithsonian Institution’s National Museum of Natural History (USNM), which curates the largest sample globally of wild orangutan skeletons with associated skins and recorded weights-at-death. Body mass was estimated within the USNM subsample using recorded weight-at-death, femoral head diameter, bi-iliac breadth, and long bone CSA. Recorded weights-at-death derive from orangutans held in the Smithsonian’s Abbott collection and have been used extensively in previous studies (Lyon 1907, 1908, 1911; Eckhardt 1975; Markham and Groves 1990; Smith and Jungers 1997; Burgess et al. 2018). Femoral head diameter was measured superoinferiorly using sliding calipers. Bi-iliac breadth was measured using a tape measure with both innominate bones articulated with the sacrum. Long bone CSA was measured from medical CT scans (130 kV, 0.63 mm slice thickness, and 250–500 micron pixel range). Using maximum length, the slice midway (50%) along the shaft was selected except for the humeri, for which the slice was selected more distally (40%) from the distal end (see Ruff et al. 2013). CSAs were measured from these slices using the BoneJ plugin in ImageJ (Doube et al. 2010). Box plots were produced using R (RCoreTeam 2013).

Descriptive statistics include means, ranges of variation, and 95% confidence intervals around the mean using a bootstrap (1000 iterations) (Tables 3 and 4). ANOVA with a Tukey post-hoc test as well as a Dunn (1964) Kruskal–Wallis multiple comparison adjusted with the Holm method were used to evaluate whether the means and medians, respectively, were the same among groups. Sample sizes for the statistical analyses merged likely-flanged and confirmed-flanged males into one flanged male group, and likely-unflanged and confirmed-unflanged males into one unflanged male group.

**Table 3.** Means, confidence intervals, ranges of variation, and post-hoc pairwise comparison *P* values for long bone lengths by category for a subsample of Smithsonian Institution individuals*

Category^1^	Weight (kg)			Femoral head diameter (mm)			Bi-iliac breadth (cm)			Femoral cross-sectional area (mm²)		
	*n*	mean^2^	ROV^3^	*n*	mean	ROV	*n*	mean	ROV	*n*	mean	ROV
Adult flanged male	6	83.8	72.5–90.6	7	38.8	35.3–40.9	7	29.2	27.6–32	7	328.7	299.7–374.6
		(79.3–89.5)			(37.6–40.3)			(27.9–30.3)			(311.7–342.50)	
Adult unflanged male	1	54.4	NA	2	35	34.5–35.6	2	25.3	23.4–27.0	2	273.1	266.8–279.5
		NA			(34.5–35.6)			(23.5–27.0)			(266.8–279.5)	
Adult female	13	36.7	31.7–45	14	30.7	28.1–32.5	11	23.84	22.1–27.0	14	209.2	178.5–249.6
		(34.3–38.7)			(30.1–31.4)			(22.9–24.6)			(197.1–220.8)	
Young adult unflanged male	2	31.7	29.5–34.0	3	30.7	27.2–32.9	2	20.9	20.8–21.0	3	173.6	161.2–181.4
		(29.4–34.0)			(28.5–34.2)			(20.8–21.0)			(165.7–186.0)	
Category comparisons^5^	Post-hoc *P* val^4^			Post-hoc *P* val			Post-hoc *P* val			Post-hoc *P* val		
Adult flanged male and adult female	<0.001 | 0.004			<0.001 | <0.001			<0.001 | 0.004			<0.001 | 0.002		

* The Smithsonian Institution National Museum of Natural History (USNM) holds the largest sample of orangutans globally with skins, skulls, and skeletons in association

1 The likely-flanged and confirmed-flanged are collapsed into one group, and the adult likely-unflanged and adult confirmed-unflanged are collapsed into one group

2 The numbers is the parentheses are the bootstraped (1000 iterations) 95% confidence intervals of the mean

3 ROV = range of variation, calculated as the minimum value to the maximum value

4 The *P* values at left are from the ANOVA Tukey post-hoc pairwise comparisons, those at right are from the Kruskal–Wallis Dunn post-hoc pairwise comparisons adjusted with the Holm method

5 The first category listed is the one that is either equal to or larger than the second category

**Table 4.** Means, confidence intervals, ranges of variation, and post-hoc pairwise comparison *P* values for long bone lengths by category for the entire sample

Category^1^	Femur length (cm)			Humerus length (cm)			Tibia length (cm)				Radius length (cm)		Ulna length (cm)		
	*n*	mean^3^	ROV^4^	*n*	mean	ROV	*n*	mean	ROV	*n*	mean	ROV	*n*	mean	ROV
Adult flanged male	37	29.1	24.3–36.4	39	37.1	31.7–44.3	37	25.4	23.0–31.2	44	37.2	31.9–43.5	44	38.7	33.0–45.3
		(28.3–29.7)			(36.3–37.9)			(24.8–25.9)			(36.5–37.9)			(38.0–39.4)	
Adult unflanged male	14	28.1	24.8–33.0	14	35.7	33–37	12	24.1	21.8–26.2	14	36.5	33.2–39.5	11	37.8	34.5–31.4
		(27.0–29.1)			(34.9–36.6)			(23.4–24.8)			(35.5–37.6)			(36.4–39.1)	
Adult female	44	25.3	23.0–28.0	44	32.5	29.3–35.9	42	21.8	19.3–23.8	45	32.8	29.6–35.5	44	33.8	30.3–36.7
		(24.9–25.64)			(32.0–32.9)			(21.4–22.1)			(32.4–33.2)			(33.3–34.3)	
Young adult likely-unflanged male	5	24.6	22.0–26.2	5	32.2	28.4–34.2	5	21.1	18.3–22.5	5	31.5	28.7–34.3	5	32.5	29.7–35.3
		(23.5–25.9)			(30.8–34.2)			(20.0–22.6)			(29.9–33.0)			(31.0–34.1)	
Category comparisons^2^	Post-hoc *P* val^5^			Post-hoc *P* val			Post-hoc *P* val			Post-hoc *P* val			Post-hoc *P* val		
Adult flanged male and adult female	**<0.001** | **<0.001**			**<0.001** | **<0.001**			**<0.001** | **<0.001**			**<0.001** | **<0.001**			**<0.001** | **<0.001**		
Adult unflanged male and adult female	**<0.001** | **<0.001**			**<0.001** | **<0.001**			**<0.001** | **<0.001**			**<0.001** | **<0.001**			**<0.001** | **<0.001**		
Adult female and young adult unflanged male	0.88 | 0.69			1 | 0.97			0.79 | 0.68			0.43 | 0.79			0.52 | 0.87		
Adult flanged male and adult unflanged male	0.34 | 0.70			0.16 | 0.63			**0.03** | 0.21			0.64 | 0.53			0.54 | 0.48		
Adult flanged male and young adult unflanged male	**<0.001** | **<0.001**			**<0.001** | **<0.001**			**<0.001** | **<0.001**			**<0.001** | **<0.001**			**<0.001** | **<0.001**		

1 The likely-flanged and confirmed-flanged are collapsed into one group, and the adult likely-unflanged and adult confirmed-unflanged are collapsed into one group

2 The first category listed is the one that is either equal to or larger than the second category

3 The numbers is the parentheses are the bootstraped (1000 iterations) 95% confidence intervals of the mean

4 ROV = range of variation, calculated as the minimum value to the maximum value

5 The *P* values at left are from the ANOVA Tukey post-hoc pairwise comparisons, those at right are from the Kruskal–Wallis Dunn post-hoc pairwise comparisons adjusted with the Holm method. Values <0.05 are in bold

## Results

Within the study sample, there were 45 individuals identified as flanged males, 46 as adult females, and 20 as unflanged males (Table 1). Based on third molar eruption and root development, all 32 flanged males were classified as adults, whereas among the 20 unflanged males, 15 and 5 were classified as adults and young adults, respectively. Among the 65 adult and young adult males, 17 and 3 were confirmed (i.e., based on associated skins) as flanged and unflanged, respectively, while 28 and 17 were identified as likely (i.e., based on long bone epiphyseal development) flanged and unflanged, respectively. Within the USNM subsample of 27 individuals, 7 were confirmed as flanged males, 3 were confirmed as adult unflanged males, 3 were identified as young adult likely-unflanged males, and 14 were adult females.

**Fig. 1:**
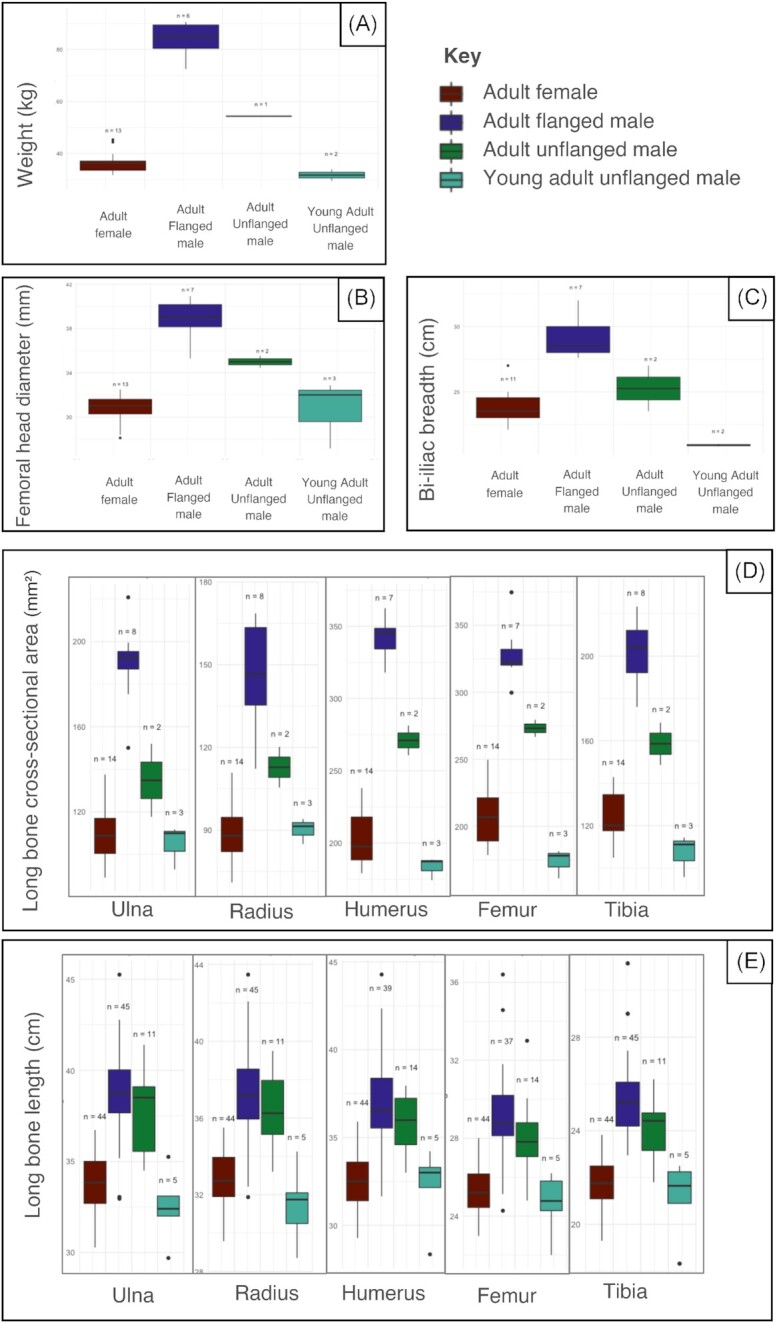
Box plots of weight-at-death (A), femoral head diameter (B), bi-iliac breadth (C), and long bone CSA (D) for the subsample of 27 orangutans. Box plots of maximum long bone length for the humerus, radius, ulna, femur, and tibia in the total sample (*n* = 111) (E).

Descriptive statistics for the USNM subsample are shown in Table 3 and for the entire sample in Table 4. In terms of weight-at-death (Fig. 1A), one of the two young adult unflanged males (34.0 kg) overlapped with the range for adult females (31.7–45.0 kg), while the other fell just outside the lower end of the range (29.5 kg). The single adult unflanged male (54.4 kg) was intermediate between the adult female range and that of flanged males (79.28–90.6 kg). All three young adult unflanged males had femoral head diameters (27.2–32.9 mm) that overlapped with the adult female range (28.1–32.50 mm), while the two adult unflanged males had values (34.5–35.6 mm) intermediate between those of females and flanged males (28.1–32.5 mm and 35.3–40.9 mm, respectively) (Fig. 1B). For bi-iliac breadth (Fig. 1C), the two young adult unflanged males (20.8–21.0 cm) overlapped with adult females (22.1–27.0 cm), while the two adult unflanged males (23.5–27.0 cm) were intermediate between adult females and flanged males (22.1–27.0 cm and 27.6–31.0 cm, respectively). In terms of long bone CSA, adult unflanged males fell intermediate between flanged males and adult females for all five measured long bones. For example, the two adult unflanged males had femoral CSAs of 266.8–279.5 mm² compared with ranges of 178.5–249.6 mm² for adult females and 299.7–339.3 mm² for flanged males (Fig. 1D). For all three arm bones, the young adult unflanged males had CSAs that overlapped with adult female ranges, but for tibial and femoral CSAs, the ranges for unflanged males and adult females overlapped only at the extremes. Three young adult unflanged males had femoral CSAs (161.2–181.4 mm²) that overlapped with the lower end of the range for adult females (178.5–249.6 mm²) (Fig. 1D).

Measures related to stature—the maximum lengths of the humerus, ulna, radius, femur, and tibia—were estimated separately within the total sample (*n* = 111). The four young adult unflanged males tended to overlap with the adult female samples, while adult confirmed-unflanged and likely-unflanged male samples tended to overlap with the range for the flanged male samples. For all long bones, the young adult unflanged male means did not significantly differ from those of the adult females (all *P* > 0.05). Conversely, the adult unflanged male means significantly differed from the adult female means for all long bones (all *P* < 0.001). Comparisons of the adult unflanged male sample with those of the flanged males were more variable. The adult unflanged male means did not significantly differ from those of flanged males for femur length (*P* = 0.21), radius length (*P* = 0.50), ulna length (*P* = 0.33), or humerus length (*P* = 0.10) but did significantly differ in tibia length (*P* < 0.01). All adult unflanged males had humerus, femur, radius, and ulna lengths that overlapped with the range for flanged males, whereas only nine of the thirteen had tibial lengths that overlapped with the flanged male range. All comparisons between adult females and flanged males were significantly different (*P* < 0.01).

## Discussion

All measurements that estimated body mass within the USNM subsample (recorded weight-at-death, femoral head diameter, bi-iliac breadth, and CSA) showed a pattern whereby young adult unflanged males overlapped with the adult female range, whereas adult unflanged males had values intermediate between those of flanged males and adult females (Table 3; Fig. 1A–D). Measurements related to stature (i.e., long bone lengths) for the entire sample showed a similar pattern, whereby young adult unflanged males did not significantly differ from adult females but did differ significantly from flanged males (Table 4; Fig. 1E). Diverging from the pattern observed for mass, mean long bone lengths significantly differed between adult unflanged males and adult females but did not significantly differ between flanged males and adult unflanged males (all except tibia length, in which the means differed but not the medians; Table 4). All comparisons between the means of adult females and flanged males were significantly different.

Evolutionary explanations for sex differences in the skeleton have been historically centered around male-male competition (Darwin 1871; Trivers 1972; Clutton-Brock and Harvey 1977; Leutenegger and Kelly 1977), including for orangutan male body sizes (Leutenegger and Kelly 1977; Mitani 1985; Rodman and Mitani 1987; Masterson and Leutenegger 1992; Uchida 1992; Leigh and Shea 1995). Among orangutans, flanged males are largest in size likely due to selection pressures related to intense competition for mates. Flanged males are known for calling and waiting for females to seek them out as mates, a strategy called “sit and wait,” and then are more likely to mate with parous females (Galdikas 1985b; Schürmann and van Hooff 1986; Utami et al. 2002; Utami Atmoko et al. 2009a; Banes et al. 2015 ). Flanged males defend the area around them, are aggressive with one another ( Mitani 1985; Galdikas 1985a; Masterson and Leutenegger 1992; Utami Atmoko et al. 2009b ), and appear to be the preferred mating choice for females (Knott et al. 2010).

They are also socially dominant to adult unflanged males, which have been observed fleeing from flanged males and being supplanted in fruit trees; however, flanged males have been observed to be less aggressive with adult unflanged males than with other flanged males (Mitani 1985; Galdikas 1985a; Masterson and Leutenegger 1992; Utami et al. 1997; Utami Atmoko et al. 2009a; Knott and Kahlenberg 2011; Banes et al. 2015). In contrast, it has been argued that adult unflanged males are under lower mate choice selection (Utami Atmoko and van Hooff 2004). In this regard, mating attempts by adult unflanged males are resisted by females more often than are those of flanged males ( MacKinnon 1974; Galdikas 1985a; Sugardjito et al. 1987; Masterson and Leutenegger 1992; Mitra Setia et al. 2009; Utami Atmoko et al. 2009a).

Adult unflanged males have generally been expected to be much smaller than flanged males, in the range of adult females specifically, as a consequence of reduced mate competition and for the benefit of reduced aggression received from larger males (Rodman and Mitani 1987). Interestingly, our results show that this size relationship is not always supported given the distributions of various proxies for adult unflanged male body sizes compared to those of adult females and flanged males. For all measurements, flanged males were always much larger than adult females, usually around 2.3 times as large, with an adult female mean of 36.7 kg and an adult flanged male mean of 83.8 kg. In contrast, the results for measures related to mass (i.e., weight-at-death, femoral head diameter, bi-iliac breadth, and long bone CSA) show that young adult unflanged males are the size of adult females, while adult unflanged males fall between adult female and flanged male size ranges. However, in measures relating to stature (i.e., maximum long bone lengths), young adult unflanged male means did not significantly differ from those of adult females, but adult unflanged males fell on a spectrum ranging between the adult females and flanged males. Thus, the results of this study do not support the prevailing idea that all unflanged males are “female-sized.”

We discuss four possible explanations for these results and showcase the implications for discussions of sexual dimorphism in orangutans. First, sexual selection may vary across time (e.g., dominance hierarchy shifts) and space (e.g., field site), at different locations and/or periods of time, adult unflanged males may experience varying levels of male-male competition and/or female mate choice. For example, at the Kinabatangan Orang-utan Conservation Project (KOCP) in Borneo, out of 10 father-offspring pairs, only one offspring was sired by an adult unflanged male (Goossens et al. 2006), whereas at Ketambe in Sumatra, >50% of offspring (6 of 11) were sired by three unflanged males (Utami et al. 2002). While flanged males are more successful in social dominance, unflanged males increase their reproductive success during periods of rank instability (Delgado and van Schaik 2000; Utami et al. 2002; Goossens et al. 2006; Banes et al. 2015). Accordingly, four of six offspring sired by unflanged males at Ketambe occurred during “unstable periods” when the dominant flanged male was not present (Utami et al. 2002). Moreover, the earlier narrative of adult unflanged males usually forcing copulations has since been complicated by observations of flanged males also forcing copulations (Delgado and van Schaik 2000; Knott and Kahlenberg 2007; Beaudrot et al. 2009; Utami Atmoko et al. 2009a; Knott et al. 2010; Banes et al. 2015). Furthermore, unflanged males appear to have higher siring success with nulliparous females (Setia and van Schaik 2007; Mitra Setia et al. 2009; Utami Atmoko et al. 2009a ; Banes et al. 2015). It is possible mating attempts by young adult unflanged males are resisted more often by females than those of adult unflanged males because of a preference for older males. However, the difference in the level of resistance between young adults and adult unflanged males remains unknown.

Second, social status and mating success do not always translate into male reproductive success, as the outcome of male-male competition does not necessarily influence mating success nor necessarily predict reproductive success (Ellis 1995; Drea 2005) (e.g., the act of mating does not guarantee fertilization of an ovum). This lack of correlation is due to several factors, including males inability to guard females effectively and the effectiveness of subordinate males at siring a significant portion of offspring (Di Fiore 2003; Drea 2005). Thus, a straightforward relationship between male-male competition or sexual selection and sex differences in body size does not exist (Plavcan 2012). Perhaps ecological and/or environmental factors such as habitat, ecology, diet, and locomotor repertoire (Leutenegger and Kelly 1977) influence male body sizes, but if this is the case, then it is not yet fully understood.

Third, sexual selection may not adequately explain differences in adult form when an individual can experience different states. For instance, some adult males can be flanged or unflanged for their entire reproductive lifetimes, whereas others experience some amount of time in both states. While no individual has ever been observed to be flanged and then unflanged, there is plasticity in the flanged state, as “past prime” males are those whose flanges have diminished in size and are subordinate to flanged males in their prime (Knott 2009). If lifetime reproductive success for one individual can include a mix of both states, then the two states can hardly be called alternative reproductive strategies, and so it is unclear if sexual selection is the right model for this scenario. For example, a male could hypothetically remain unflanged for much of his adult life and sire no offspring, but then, at 30 years old become flanged and sire more offspring than a male that flanged at 15 and was killed at 20 by another flanged male. While siring events may be concentrated when males are in the flanged state (Scott 2021), whether males who develop flanges early in life are more successful than those who develop them later is not clear. Additionally, more potential male states exist beyond flanged or unflanged, since some orangutans are in the process of flanging while others are past prime (Knott 2009).

Finally, the rate and duration of the period of growth and development (i.e., ontogeny) may provide a more robust explanation for body size differences in adult male orangutans. In humans, adult males are, on average, taller than adult females because the onset of puberty occurs earlier in females (Dunsworth 2020). The increase in estrogen during puberty causes epiphyseal closure and cessation of linear growth for all humans; since biological males on average have a later onset of puberty than biological females and thus a longer growth period, they are on average taller (Šešelj et al. 2012; Dunsworth 2020).

This pattern is also seen in the growth trajectories of female and male great apes (Dunsworth 2020). Large sex differences in adult orangutan body sizes have been attributed to indeterminate or unlimited male growth, whereby males in captivity have been observed to increase in weight throughout their lifespan (Leigh and Shea 1995). If this is the case, then the longer adult unflanged males grow, the larger and taller they are expected to become. Thus, ontogenetic plasticity could explain the range of body sizes in this study, even across the wide range of dental wear stages observed. Additionally, in relation to one another, flanged and unflanged males have similar canine heights but vary considerably in body size. This variation in body size may be due to fluctuations in conditions over an individual's lifespan that result in a more plastic response compared to that for canine development.

Previous claims that adult unflanged males are “female-sized” almost certainly stem from the fact that adolescent males are often “female-sized,” although our results show that males who remain unflanged well into their adulthoods are larger than their young adult male counterparts. Of course, the four proposed possible explanations discussed herein are not mutually exclusive, and each could be contributing to the observed variation in adult male orangutan body sizes. Future research could provide further information on the paternity success of flanged and unflanged males conditioned on age and length of time spent in the unflanged state. Body size data are needed from wild counterparts using noninvasive techniques such as photogrammetry that take into account the age of the unflanged males, sampling a number of ages.

## Conclusion

In this study, we measured the largest sample of wild adult unflanged males yet assembled and distinguish between the unflanged males in earlier adulthood (young adult unflanged males) who remain without secondary sex characteristics compared to those who remain without flanges later into their adulthood (adult unflanged males). Measures of body size for young adult unflanged males had a distribution that overlapped with that of females in this sample, whereas the range of body size for adult unflanged males was intermediate between that of adult females and adult males. We placed these results in the context of the evolution of sexual dimorphism in body size in orangutans. Our findings are consistent with the proposed mechanism that some adult unflanged males may have higher male-male competition and/or female choice in certain times or locations, but may also be consistent with the idea that sexual selection is not the only driver of male body size in orangutans that is worth considering.

These findings demonstrate that orangutan body sizes do not fit neatly into two types by sex, large and small, but rather are situated on a spectrum. The term sexual dimorphism is defined as the systematic difference in form between males and females (Mesnick and Ralls 2018). We do not make the case for the term sexual trimorphism (three types by sex) because the same individuals may have a body mass between that of females and flanged males while being similar to, or larger than, flanged males for stature. The ranges observed for stature proxies were not quite the same as those for mass, and this is even further complicated by the fact that individuals fall in a spectrum within those ranges. Therefore, researchers should, at minimum, clearly specify which trait they are referring to when using the term “sexual dimorphism” to describe orangutans, as not all traits in these Asian great apes fit expectations of dimorphism. More importantly, a shift toward using terminology that better captures the ranges of variation exhibited is clearly warranted. A number of terms or phrases have been proposed as alternatives to sexual dimorphism that would capture the range of body shapes and sizes, including sex differences (Dunsworth 2020; Štrkalj and Pather 2021), sex polymorphism (Astorino 2019), sexual difference (Irigaray 1993; Grosz 2012), or sex diversity and variation. Overall, the term sexual dimorphism lacks specificity and is not especially helpful in the case of orangutan body sizes, requiring that an alternative and more evolutionarily informative phrase should be utilized.

## Data Availability

Raw data are available from the corresponding author upon reasonable request.

## Notes

From the symposium “Sexual diversity and variation” presented at the annual meeting of the Society for Integrative and Comparative Biology, in Austin, Texas, January 4, 2023.
